# Perceived Barriers and Facilitators of Use of Artificial Intelligence in Eating Disorder Care: A Commentary on Linardon et al. (2025)

**DOI:** 10.1002/eat.24426

**Published:** 2025-03-24

**Authors:** Gemma Sharp

**Affiliations:** ^1^ School of Psychology University of Queensland Brisbane Queensland Australia; ^2^ Department of Neuroscience Monash University Melbourne Victoria Australia

**Keywords:** artificial intelligence, barriers, chatbot, digital health, eating disorders, ethics, facilitators

## Abstract

Artificial intelligence (AI) has the potential to revolutionize mental health care, including for eating disorders, but there are still a number of concerns focused on ethics, governance, and regulation. As the authors found in their preliminary survey study involving mental health clinicians and people experiencing eating disorder symptoms, there was support and recognition of the benefits of AI tools in eating disorder care. However, participants also had concerns surrounding issues like data privacy, governance, information accuracy, and therapeutic rapport. From our own research involving the development of multiple AI tools, particularly chatbots, to assist people experiencing eating disorders and their loved ones, we suggest that these perceived barriers can be overcome with thoughtful and comprehensive codesign with multidisciplinary teams following ethical frameworks for AI and digital technologies. In this way, we can optimally mitigate the risk of using AI tools while still offering the most advanced technologies to treat eating disorders.

1

Artificial intelligence (AI) is broadly defined as a technology that enables computers to simulate human functions like learning, problem‐solving, and decision‐making. With mental health care systems across the world overwhelmed with demand, AI has the potential to revolutionize mental health care systems and strategies such as optimized early detection of illness, more personalized therapeutic offerings, and even AI‐driven therapeutic assistants. Despite these potentially exciting possibilities, there are considerable ethical concerns, regulatory and governance considerations, and the need for ongoing research, evaluation, and development. With eating disorders representing some of the most fatal psychiatric illnesses, the concerns are possibly even greater for the integration of AI methodologies into routine eating disorder care. For these reasons, the preliminary online survey study conducted by Linardon et al. (2025), with mental health clinicians and people experiencing eating disorder symptoms, investigating barriers and facilitators to the use of AI provides some useful insights.

In the study of 116 clinicians and 155 community participants, the authors specifically examined use of and attitudes toward AI, benefits and risks, and perceived task performance compared to humans (Linardon et al. 2025). Surprisingly, only 59% of clinicians and 18% of community participants reported prior use of AI tools in their practice or to learn more about or manage symptoms of eating disorders, respectively. ChatGPT was the most used tool for both groups. These findings suggest a potential lack of awareness, particularly from the clinicians, of the preexisting integration of AI into their routine clinical management software/technologies, as appointment scheduling, billing, clinical note creation, and so forth employ elements of AI. Therefore, the more accurate answer here is likely to be closer to 100% for clinicians. In our own research focused on AI implementation with clinicians and people with lived eating disorder experience, we have seen some similar misunderstandings of the breadth of AI usage (e.g., Beilharz et al. 2021; Sharp et al. 2025). Although, Linardon et al. (2025) did define AI in their survey (“… AI is focused on creating systems that can perform tasks that typically requires human‐level intelligence, such as problem‐solving, decision‐making, and reasoning …”) perhaps the participants found this description quite abstract. Indeed, it seemed like ChatGPT, as a more concrete example of an AI tool, was the point of reference for most participants.

It is possible that this rather abstract definition of AI also guided participant responding for the rest of the survey and is a limitation of this research. Indeed, when participants were asked whether they believed AI would improve outcomes for people with eating disorders, around 58% of clinicians and 63% of community participants reported being “undecided” (Linardon et al. 2025). Furthermore, approximately 20%–30% of clinicians and community participants answered with “neither disagree/agree” when presented with a range of potential benefits of AI tools (e.g., convenience, anonymity). Such findings possibly suggest that at least some participants provided a default neutral answer as they could not get a strong sense of the specific AI tools that would deliver those services/benefits. In our research, we have found that providing and demonstrating specific examples, especially at the earliest stages of AI tool codesign processes, elicits more certain feedback from participants (e.g., Beilharz et al. 2021; Sharp et al. 2025). Nevertheless, the majority of participants answered “somewhat agree/strongly agree” for the presented benefits of AI (Linardon et al. 2025), suggesting that there is support for future integration of AI tools in eating disorder care.

Both groups of participants were clearly concerned about the risks of AI presented in the study, such as data privacy, regulatory challenges, and the presentation of inaccurate information. These are commonly raised concerns by clinicians and people with lived eating disorder experience in our experience developing AI tools (Sharp et al. 2025, 2023). The field of eating disorders also has an additional unfortunate incident commonly cited by key stakeholders when AI tools are discussed in Tessa chatbot providing inappropriate weight loss and dieting advice in the United States in June 2023 (e.g., Sharp et al. 2025 and references therein). Given that ChatGPT was seemingly the most common AI tool point of reference for participants, it is likely that at least some participants were prompted to think of this highly publicized chatbot incident when completing the survey.

Clinicians and community participants alike also expressed concerns about whether AI tools could provide empathic care, build therapeutic rapport, and manage crisis situations as well as or better than a human. Again, these are very understandable and commonly expressed concerns from our research experiences (e.g., Beilharz et al. 2021; Sharp et al. 2025). None of these concerns should necessarily stop progress, they just need to be factored into the design and implementation of the tools. For example, we have found that users are generally keenly aware that they are conversing with an AI tool and so empathy from such a tool sounds inauthentic (e.g., a chatbot saying “I know how you feel” when a machine does not have feelings). From our experience, users want AI tools to present factual information while having an authentic validating tone (e.g., “A lot of people find it hard to take the first step for seeking help so it is great that you are here”) (e.g., Beilharz et al. 2021; Sharp et al. 2025).

Managing crises is another situation where we have codesigned solutions with key stakeholders that have been and continue to be used effectively throughout the world (e.g., versions of JEM (TM) chatbot [formerly KIT (Beilharz et al. 2021)] have been freely accessible in Australia and North America since 2020). It is important that the AI tool user be made aware of the limitations of the tool from the outset of use and be provided with crisis support contact details so they can connect with a human support service if they wish. Furthermore, in our more recent work with ED ESSI (TM), a chatbot that provides eating disorder‐focused waitlist support, we codesigned a risk detection system with people with a lived experience and clinicians whereby the chatbot conversation stopped when medical and/or psychiatric risk was detected, the user was encouraged to seek immediate medical care, and an alert was sent to the treating team for them to follow up (Sharp et al. 2025). Importantly, the user agreed to this risk assessment system at the start of using the tool, and the system worked effectively in our randomized controlled trial (results under peer review).

We are in a time where AI's capabilities are in advance of governance and regulation. The AI tools from our team referenced above are all rule‐based (i.e., can only give preprogrammed answers) to ensure the highest level of safety. This was deemed particularly desirable in our codesign research (Sharp et al. 2025) so as to best avoid another incident like Tessa chatbot's provision of inappropriate weight loss and dieting advice. However, as found by Linardon et al. (2025), clinicians and community members are using generative AI tools like ChatGPT for assistance with the delivery of clinical care and receiving this care. So how do we maintain a high level of safe, private, accurate, evidence‐based care, but provide users with the most technologically advanced experiences possible? It is something those of us in the field are challenged by every day as we continue to develop and test more advanced tools using generative AI.

We cannot completely eliminate risk, just as with humans delivering care to people with eating disorders, but we can engage in comprehensive and ethical design of AI tools to best mitigate these risks. The ideal team for this design work includes mental health clinicians, researchers, developers, individuals with lived eating disorder experience, and ethicists. Each team member brings expertise integral to the success of an AI tool (Sharp et al. 2023). There exist also ethical frameworks for AI and digital technologies to help guide us through the domains/factors that should be considered in design and implementation—physical (dignity, well‐being, safety, and sustainability), cognitive (intelligibility, accountability, fairness, and autonomy), information (privacy and security), and governance (financial, economic, individual, and societal impacts) (Ashok et al. 2022; Sharp et al. 2023).

In conclusion, the preliminary survey study by Linardon et al. (2025) provides a snapshot of the perceptions of clinicians and community members on AI use in eating disorder treatment. Even with a rather abstract definition of AI whereby participants may not have been entirely certain of the form of the AI tool(s) to which they were invited to respond, clinicians and people with eating disorder symptoms commonly endorsed potential benefits. Understandably, they had concerns too surrounding issues like data privacy, governance, information accuracy, and therapeutic rapport. However, with thoughtful and comprehensive codesign following ethical frameworks, these issues can potentially be overcome to allow people with eating disorders and their loved ones to experience the full benefit of AI capabilities in their care.

## Author Contributions

**Gemma Sharp:** conceptualization, writing – original draft, writing – review and editing.

## Conflicts of Interest

The author declares receiving funds for the licensing of multiple conversational AI technologies to not‐for‐profit organizations.

## Data Availability

Data sharing is not applicable to this article as no datasets were generated or analyzed during the current study.

## References

[eat24426-bib-0001] Ashok, M. , M. Madan , A. Joham , and U. Sivarajah . 2022. “Ethical Framework for Artificial Intelligence and Digital Technologies.” International Journal of Information Management 62: 102433. 10.1016/j.ijinfomgt.2021.102433.

[eat24426-bib-0002] Beilharz, F. , S. Sukunesan , S. L. Rossell , J. Kulkarni , and G. Sharp . 2021. “Development of a Positive Body Image Chatbot (KIT) With Young People and Parents/Carers: Qualitative Focus Group Study.” Journal of Medical Internet Research 23, no. 6: e27807. https://www.jmir.org/2021/6/e27807/. 34132644 10.2196/27807 PMC8277317

[eat24426-bib-0003] Linardon, J. , C. Liu , M. Messer , Z. McClure , C. Anderson , and H. K. Jarman . 2025. “Current Practices and Perspectives of Artificial Intelligence in the Clinical Management of Eating Disorders: Insights From Clinicians and Community Participants.” International Journal of Eating Disorders 58, no. 4: 724–734. 10.1002/eat.24385. 39829089 PMC11969028

[eat24426-bib-0004] Sharp, G. , B. Dwyer , J. Xie , et al. 2025. “Co‐Design of a Single Session Intervention Chatbot for People on Waitlists for Eating Disorder Treatment: A Qualitative Interview and Workshop Study.” Journal of Eating Disorders 13, no. 1: 46. 10.1186/s40337-025-01225-x. 40069853 PMC11899673

[eat24426-bib-0005] Sharp, G. , J. Torous , and M. L. West . 2023. “Ethical Challenges in AI Approaches to Eating Disorders.” Journal of Medical Internet Research 25: e50696. https://www.jmir.org/2023/1/e50696. 37578836 10.2196/50696 PMC10463082

